# Intestinal parasitic infections in a community from Pampa del Indio, Chaco (Argentina) and their association with socioeconomic and environmental factors

**DOI:** 10.1371/journal.pone.0285371

**Published:** 2023-06-29

**Authors:** Carlos Matias Scavuzzo, Cintia Delgado, Marcia Goy, Favio Crudo, Ximena Porcasi, María Victoria Periago

**Affiliations:** 1 Fundación Mundo Sano, Buenos Aires, Argentina; 2 Instituto de Altos Estudios Espaciales Mario Gulich, Universidad Nacional de Córdoba, Comisión Nacional de Actividades Espaciales, Córdoba, Argentina; 3 Consejo Nacional de Investigaciones Científicas y Técnicas (CONICET), Buenos Aires, Argentina; 4 Hospital Dr. Dante Tardelli, Pampa del Indio, Chaco, Argentina; University of Uyo, NIGERIA

## Abstract

Neglected tropical diseases are a group of 20 disabling diseases, which, in particular, are the most common chronic infections in the most vulnerable people. This study aimed to characterize the infection by intestinal parasites (IPs) in dwellings from a peri-urban neighborhood in Pampa del Indio, Chaco (Argentina), and its association with socioeconomic and environmental variables. Single stool samples were collected from all individuals older than 1 year through household visits and processed using coprological sedimentation and flotation techniques. Standardized questionnaires were used at the household level to collect socio-economic information. Environmental variables were obtained from the Planetscope image, Landsat 8 images and remote sensors, while land-use layers were obtained through the use of a maximum likelihood algorithm. Stool samples were provided by 314 individuals. The prevalence of IPs found was 30.6% (n = 96), with a predominance of *Giardia lamblia* (12.7%, n = 40) and *Hymenolepis nana* (7.6%, n = 24). The only soil-transmitted helminth found was *Strongyloides stercoralis* with a 2.5% prevalence (n = 8). Individuals of adult age (> 18 years) were 0.65 times less likely to present parasitic infections with respect to children and adolescents. The only environmental variable that was closely associated with the presence of IPs, was the Normalized Difference Water Index (NDWI), a measure of humidity; being higher around houses with positive individuals. Most of the IPs found in this study were of water-borne transmission and those transmitted directly from person-to-person, therefore fecal contamination is present. We believe that the low prevalence of STH in this area, which requires a passage through the soil, is related to the environmental characteristics, which are unsuitable for the development/permanence of the infective stages of these parasites. The geospatial data and tools used herein proved to be useful for the study of the relationship between the different factors that influence the presence of IPs in a community, from an eco-health approach.

## Introduction

Neglected tropical diseases (NTDs) are a group of 20 disabling diseases recognized by the World Health Organization (WHO), which are the most common chronic infections in the most vulnerable people [1–4]. These include soil-transmitted helminths (STH), such as *Ascaris lumbricoides*, *Trichuris trichiura*, and hookworm, which are intestinal parasites (IPs). Other IPs are the protozoans, like *Giardia intestinalis*, *Entamoeba histolytica*, and *Cryptosporidium* spp. [5]), which can also limit the health and nutritional status of their hosts, leading to iron deficiency anemia, growth and cognitive retardation [6–11]. A separate but equally important case to consider is the STH *Strongyloides stercoralis*, given its worldwide prevalence and burden [12]. Although this species was not originally contemplated within the control strategy designed by the WHO for STH, due to specific requirements for diagnosis and treatment; it was included in 2021 [13].

Evidence shows that NTDs, poverty, and certain combinations of ecological, social, political, and economic determinants are strongly correlated, and these factors explain the emergence of hotspots worldwide, especially in developing countries and vulnerable communities [14–16]. Although IPs as a group are not included in the list of NTDs, only STHs are, other IPs have also been found to be associated with similar determinants. Research conducted among urban, peri-urban, and rural populations in Argentina have observed different prevalence rates of IPs, ranging from 0.5 to 88.9%, according to socioeconomic level, sanitary and environmental conditions, and water supply [17–32], with a predominance of STHs in northeast and northwest provinces of the country. Nonetheless, despite having STH prevalence greater than 20% in some areas of the country, mass drug administration (MDA) programs were implemented for a very short period of time (2005–2007) and then discontinued [33].

An in-depth analysis of the complex systems involved in infectious diseases, as well as their causes and consequences, requires more integrative paradigms, such as the ecosystem approach to human health (eco-health or one-health), which incorporates ecological, biological, and social factors as well as their possible interactions [34]. One tool that is useful for this type of approach is the concept of landscape epidemiology, which is widely used in the area of geomatics and remote sensing (RS) to refer to environmental conditions such as land cover, land use, and composition, climatic and geographic characteristics, among others [35–38]. A great variety of environmental conditions can be readily obtained from RS (satellite images and products), making them useful in various fields, such as the preparation of risk maps to guide health effectors and prioritize resources for different infectious diseases [39–43].

Cross-sectoral collaboration, including education, nutrition, and agriculture, has strengthened the control of certain infectious diseases [44]. Working to overcome the impact of many infectious diseases, especially those that affect children, represents a largely untapped development opportunity to alleviate poverty for many populations and thus have a direct impact on achieving international collaborative agendas such as the Sustainable Development Goals (SDGs) or the new roadmap for NTDs [44, 45]. The province of Chaco in Argentina is part of the Gran Chaco Ecoregion, which is considered a hotspot for NTDs, especially Chagas Disease (ChD) [14, 46]. The city of Pampa del Indio has been extensively studied with respect to ChD [47], with a focus on vector surveillance and control, but there is a lack of published studies on IPs and deworming campaigns have not been implemented in this area.

Given the relationship between the environment, humans, certain social determinants and the transmission of IPs, this study aims to identify the IP infections present in individuals from a neighborhood of Pampa del Indio, Chaco, Argentina; and determine if there are any associations between the presence of IPs and different socioeconomic and environmental variables. For this purpose, we have collected socioeconomic data through a household questionnaire, parasite presence through the analysis of fecal samples; and land-use and environmental indexes derived from remote sensed data that potentially play a role in the presence of IPs as determined in previous studies [24, 43, 47–51].

## Materials and methods

### Study area

Fieldwork was conducted in a peri-urban neighborhood of Pampa del Indio (Lat: -26.0473; Long: -59.9416), Chaco province, northeastern Argentina, located in the transition between the humid and dry Chaco region. The municipality was inhabited by approximately 22,000 people in 2013. Official records from the 2001 and 2010 decennial census indicated that the population of Pampa del Indio municipality increased markedly from 11,558 to about 18,000 people, respectively (annual population growth rate, 4.9%) [52, 53].

**Fig 1:**
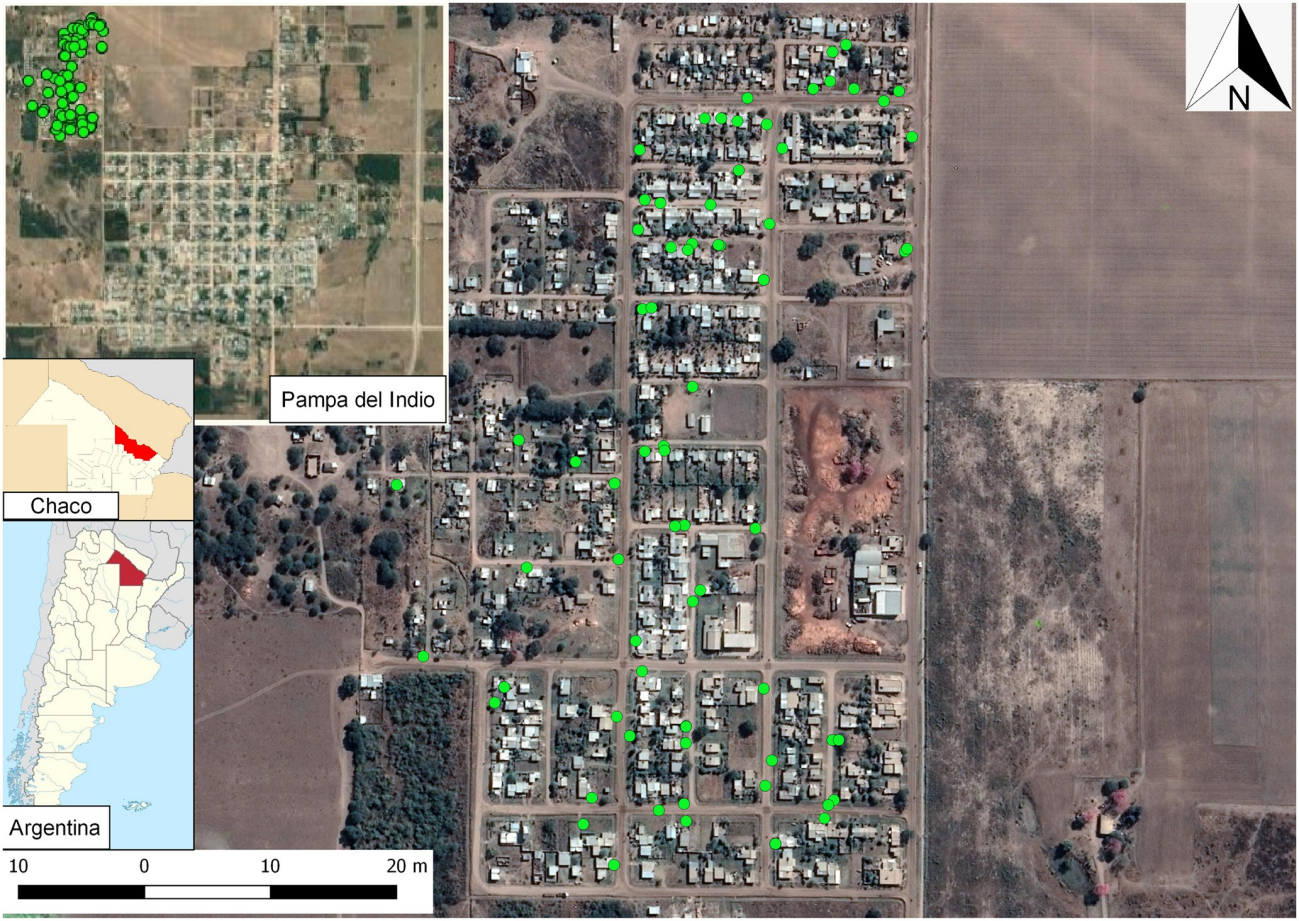
Study area in Pampa del Indio, Chaco (Argentina). The neighborhood included in this study was a peri-urban area called Parque Industrial. Map data © 2020 Google, base map obtained through QuickMapServices QGIS plugin—QGIS Geographic Information System. Open Source Geospatial Foundation Project. http://qgis.osgeo.org. Contains information from OpenStreetMap and OpenStreetMap Foundation, which is made available under the Open Database License.

The climate is currently continental, mostly warm, with more rainfall in the summer. The average annual temperature is 22.8 °C (average minimum and maximum, 16.9 and 29.3 °C). Annual rainfall has historically been 954 mm. The landscape is flat and consists mainly of a mosaic of crop patches mixed with the native dry forest that has undergone varying degrees of degradation, and occasional water bodies and swamps [54–56]. Houses included in this study are located on the periphery of Pampa del Indio, in the peri-urban neighborhood of Parque Industrial, an area surrounded by agricultural fields mixed with patches of native forest subject to varying degrees of degradation (Fig 1). Based on this characterization, this neighborhood was selected as a first step to determine the presence of IPs in the area since there was no previous data on these infections in the population.

### Study design

The study was designed as a cross-sectional study which was conducted between June 2016 and January 2017. Parque Industrial is composed of 22 blocks and houses were randomly selected assuring at least four houses from each block were included, one from each of the four sides of each block. Each house was georeferenced and characteristics of each household, as well as demographic data from each of the inhabitants, was collected through the use of a standardized questionnaire. The questionnaire was divided into different sections and covered aspects related to education, occupation, household characteristics (including source of drinking water, type of toilet, as well as construction materials) and the presence/ownership of domestic animals.

If a household declined to participate, the next household on that same side of the block was visited. Sterile and leak-proof wide-cap containers without any fixative were distributed to all the members of the families which showed interest in participating as evidenced through the signed consent/assent. During the initial visit, instructions were given on the ideal way to collect stool samples, by defecating on a clean surface (bag or paper), without contact with water, urine or dirt and then with a wooden spatula to place a good amount of sample into the container. Participants were told the team would return on the following day to retrieve the samples and that they were to be kept in a shaded area of the house. A single stool sample was collected for each individual and houses were visited at least on three occasions in order to give individuals time to produce the sample. The samples were transported in a cool box to the Clinical Analysis Laboratory of the public hospital “Dr. Dante Tardelli” of Pampa del Indio, where all the samples were analyzed macroscopically for the presence of parasitic forms and then processed by three different concentration methods including sedimentation (Telemann), salt flotation (Willis), and sugar flotation (Sheather), as previously described [57]. Infected individuals were clinically evaluated by the project physician and treatment was provided by the project following national guidelines from the National Ministry of Health [58] which indicate treatment with mebendazole or metronidazole depending on the IP.

### Ethical considerations

This study was approved by the Institutional Review Board (IRB) of the National Center of Medical Genetics (Centro Nacional de Genética Médica), Administration of Laboratories and Institutes of Health (Administración Nacional de Laboratorios e Institutos de Salud–ANLIS) “Dr. Carlos G. Malbrán” of the National Ministry of Health of Argentina (protocol approved unanimously 27 May 2016). Written informed consent was obtained from all participating adults and from a parent or guardian of every child under 16 years of age. Moreover, written assent was obtained from children between the ages of 6 and 15 years of age, inclusive.

Criteria for inclusion in the study included being older than 1 year and having signed the appropriate informed consents and assents. Exclusion criteria included working or living outside the area of study for more than a week at a time or having behavioral, cognitive or psychiatric issues that may affect the ability to understand and adhere to the study protocol. Deworming is not routinely performed in this area, therefore this was not considered as criteria for exclusion.

### Environmental characteristics

The land-use map of the study area was obtained using the Planetscope image (with a spatial resolution of 3.5 m; radiometrically and geometrically calibrated) [59]. Using a maximum likelihood algorithm, a land-use layer regarding 4 classes based on updated literature [60] and the relationship with parasitic transmission cycles was obtained. These classes (C) were: C1) bushy or thicker vegetation, C2) low and sparse vegetation, C3) bare soil, and C4) urban construction. For the image classification, training (20 pixels for each class) and validation points were obtained from Airbus images. Finally, a black and white image (in a dichotomous/binary manner) was generated for each class separately and the statistics of the neighborhoods of each house were extracted.

Additionally, several spectral indexes to characterize the environment surrounding each house were generated using Landsat 8 image collections from the Google Earth Engine platform [61, 62]. These environmental variables were calculated as the annual averages of year 2016; and are commonly used in spatial epidemiology and disease mapping [88]: Normalized Vegetation Index (NDVI) as a proxy of vegetation cover, Normalized Difference Water Index (NDWI) as a proxy of wetness, Normalized Snow Differential Index (NDSI) as a proxy for soil characteristics, and Normalized Burnt Area Index (NBRT) as a proxy for soil temperature, were included.

To extract the indices from the vicinity of each house, a buffer of 50 m around each house was used through zone statistics tools provided by Qgis software; spatial and environmental analysis was performed with open source Qgis software version 3.10 [63]. Descriptive and exploratory statistical analyses were performed with Stata software version 15.1 [64]. Moreover, in order to evaluate whether there were significant differences among means (quantitative) and proportions (categorical) between sociodemographic characteristics and the presence of intestinal parasites, a t-test was used.

### Spatial analysis

To visualize the spatial dimension of infection by IPs, the number of cases per household was represented through heat maps. Later, a spatial cluster analysis was performed with Satscan software [65] using a Bernoulli distribution model to explore the distribution of households with infected and non-infected individuals. If spatial clusters were detected, the environmental indexes obtained through RS for the households within the significant cluster were compared with those of the households outside the cluster, using independent mean tests.

To determine the association of the different variables and the presence or absence of IPs, logistic regression was used. Through initial bivariate logistic regression models, the relationship between different variables were tested in order to discard covariates and proceed with the multivariate logistic regression. Finally, a negative binomial model with robust deviancy, using the quasi maximum likelihood method was used to model the risk of infection of IPs.

## Results

A total of 127 dwellings were surveyed and 525 containers were distributed throughout the study period. Participation was 59.8%, given that 314 individuals from 107 of the households provided stool samples for analysis. IPs were found in 96 individuals (30.6%), many of them polyparasitized with more than one species (n = 35, 11.1%), not all of them pathogenic (Table 1). The only STH species found was *S*. *stercoralis*; this parasite was detected in 8 individuals (2.5%).

**Table 1:** Descriptive prevalence of intestinal parasites in inhabitants from Parque Industrial, Pampa del Indio, Chaco (Argentina), 2016–2017.

Parasitological description (N = 314 individuals) No. (%)	
Positive	96 (30.6)
Negative	218 (69.4)
**Mono-infections**	**61 (19.5)**
*Endolimax nana* ^1^	10 (3.2)
*Entamoeba coli* ^1^	13 (4.1)
*Blastocystis* spp.^1^	4 (1.3)
*Chilomastix mesnili* ^1^	3 (1.0)
*Giardia lamblia*	17 (5.4)
*Enterobius vermicularis*	3 (1.0)
*Hymenolepis nana*	8 (2.5)
*Strongyloides stercoralis*	3 (1.0)
**Double infections**	**28 (8.9)**
*E*. *coli*^1^ */ E*. *nana*^1^	3 (1.0)
*E*. *nana*^1^ */ C*. *mesnili*^1^	1 (0.3)
*G*. *lamblia / Blastocystis* spp.^1^	1 (0.3)
*G*. *lamblia / E*. *coli*^1^	3 (1.0)
*G*. *lamblia / E*. *nana*^1^	5 (1.7)
*G*. *lamblia / Hymenolepis nana*	7 (2.2)
*H*. *nana / C*. *mesnili*^1^	2 (0.6)
*H*. *nana / E*. *coli*^1^	1 (0.3)
*H*. *nana / E*. *nana*^1^	1 (0.3)
*S*. *stercoralis / C*. *mesnili*^1^	1 (0.3)
*S*. *stercoralis / E*. *nana*^1^	1 (0.3)
*S*. *stercoralis / H*. *nana*	2 (0.6)
**Triple-infections**	**7 (2.2)**
*G*. *lamblia / E*. *coli*^1^*/ E*. *nana*^1^	3 (1.0)
*G*. *lamblia / H*. *nana/ E*. *coli*^1^	1 (0.3)
*G*. *lamblia / H*. *nana/ E*. *nana*^1^	1 (0.3)
*E*. *vermicularis / H*. *nana / G*. *lamblia*	1 (0.3)
*S*. *stercoralis / G*. *lamblia*^1^/ *E*. *nana*^1^	1 (0.3)

No.: Number.

^1^Non-pathogenic species for which the patient is clinically evaluated to determine if treatment is needed, depending on medical judgment. We have included *Blastocystis* spp. as a non-pathogenic species given there is still controversy and subtyping of this protist was not an aim of the current study [47].

Data collected through the standardized questionnaires showed that only 4% of the participants were employed in agriculture, and only 7% had a vegetable garden, while the water for irrigation came from the public water supply. Almost all of the households (95%), had access to gas, either natural or bottled, although the smallest proportion was supplemented with firewood (6.5%), while all households had access to the electrical network. Also, 100% reported that the source of water for drinking, cooking, and handwashing was from the public drinking water network and none of them treated the water at home before drinking it (either by boiling or adding drops of bleach). With respect to excreta disposal, 100% had a latrine and 85% were improved with septic tank drainage. With respect to animals, 74% had at least 2 dogs and 42% had never dewormed them. It was noted that 76% of the households do not have cats. In the same sense, 98% did not raise animals and no one had animal pens.

Table 2 shows the description of the sample according to the presence or absence of IPs. Variables were analyzed both at the individual and household level, given that family income and characteristics of the household are common to all the inhabitants of each house. At the individual level, there was a higher proportion of infected children and adolescents (67.7%), 1 to 17 years of age, in comparison to adults (32.3%), older than 18 years. There was also a higher percentage of infected individuals with incomplete primary education (63.5%), although these differences were not statistically significant. From the 314 participants, 52 individuals reported having an intestinal parasitic infection on previous occasions, of which 71.2% (n = 37) were treated. With respect to the characteristics at the household level, no statistically significant differences were found between the proportions of houses with any positive cases and negative houses without infection.

**Table 2:** Sociodemographic characteristics of the study population, according to presence or absence of parasites, Parque Industrial neighborhood, Pampa del Indio, Chaco, 2016–2017.

	Complete sample	Infected	Non-infected	p-value for the difference in proportions (T-test) between individuals with and without infection
**Individual samples**	**n = 314**	**n = 96**	**n = 218**	
**Age (years), [n (%)]**				
**Children and adolescents (1–17)**	191 (60.8)	65 (67.7)	126 (57.8)	0.09
**Adults (> = 18)**	123 (39.2)	31 (32.3)	92 (42.2)	0.09
**Gender [n (%)]**				
**Female**	180 (57.3)	53 (55.2)	127 (58.3)	0.61
**Male**	134 (42.7)	43 (44.8)	91 (41.7)	0.61
**Education [n (%)]**				
**Incomplete elementary school**	209 (66.5)	61 (63.5)	148 (67.9)	0.54
**Finished elementary school**	77 (24.5)	26 (27.1)	51 (23.4)	0.30
**Finished middle school**	14 (4.5)	6 (6.3)	8 (3.7)	0.32
**No data**	14 (4.5)	3 (3.1)	11 (5.0)	
**Parasitic antecedents [n (%)]**				
**Yes**	52 (16.6)	17 (17.7)	35 (16.1)	0.96
**No**	191 (60.8)	63 (65.6)	128 (58.7)	0.96
**No data**	71 (22.6)	16 (16.7)	55 (25.2)	
**Previous treatment [n (%)]**				
**Yes**	37 (71.2)	10 (58.8)	27 (77.1)	0.54
**No**	5 (9.6)	2 (11.8)	3 (8.6)	0.54
**No data**	10 (19.2)	5 (29.4)	5 (14.3)	
**Households sampled**	**N = 107**	**N = 49**	**N = 43**	
**Household income [n (%)]**				
**Day laborer**				
**Yes**	56 (55.4)	24 (53.3)	24 (58.5)	0.71
**No**	45 (44.6)	21 (46.6)	17 (41.5)	0.72
**Social plan beneficiaries**				
**Yes**	68 (63.5)	31 (63.2)	26 (60.4)	0.82
**No**	33 (30.8)	14 (28.6)	15 (34.8)	0.72
**No data**	6 (5.6)	4 (8.1)	2 (4.6)	
**Receive retirement fund or pension**				
**Yes**	16 (14.9)	4 (8.1)	9 (20.9)	0.57
**No**	85 (79.4)	41 (83.7)	32 (74.4)	0.32
**No data**	6 (5.61)	4 (8.2)	2 (4.6)	
**Roof [n (%)]**				
**Metal sheets**	99 (92.5)	44 (89.8)	40 (93.2)	0.57
**Adobe and wood**	8 (7.4)	5 (10.1)	3 (6.9)	0.87
**Floor [n (%)]**				
**Concrete**	71 (66.3)	33 (67.5)	29 (67.4)	0.99
**Concrete and adobe**	21 (19.6)	9 (18.4)	6 (13.9)	0.81
**Ceramic**	9 (8.4)	3 (6.1)	6 (13.9)	0.72
**No data**	6 (5.6)	4 (8.2)	2 (4.6)	
**Wall [n (%)]**				
**Adobe and wood**	21 (19.8)	9 (18.3)	6 (14.3)	0.83
**Bricks**	79 (74.5)	36 (73.5)	34 (80.9)	0.46
**No data**	6 (5.7)	4 (8.2)	2 (4.8)	

**Fig 2:**
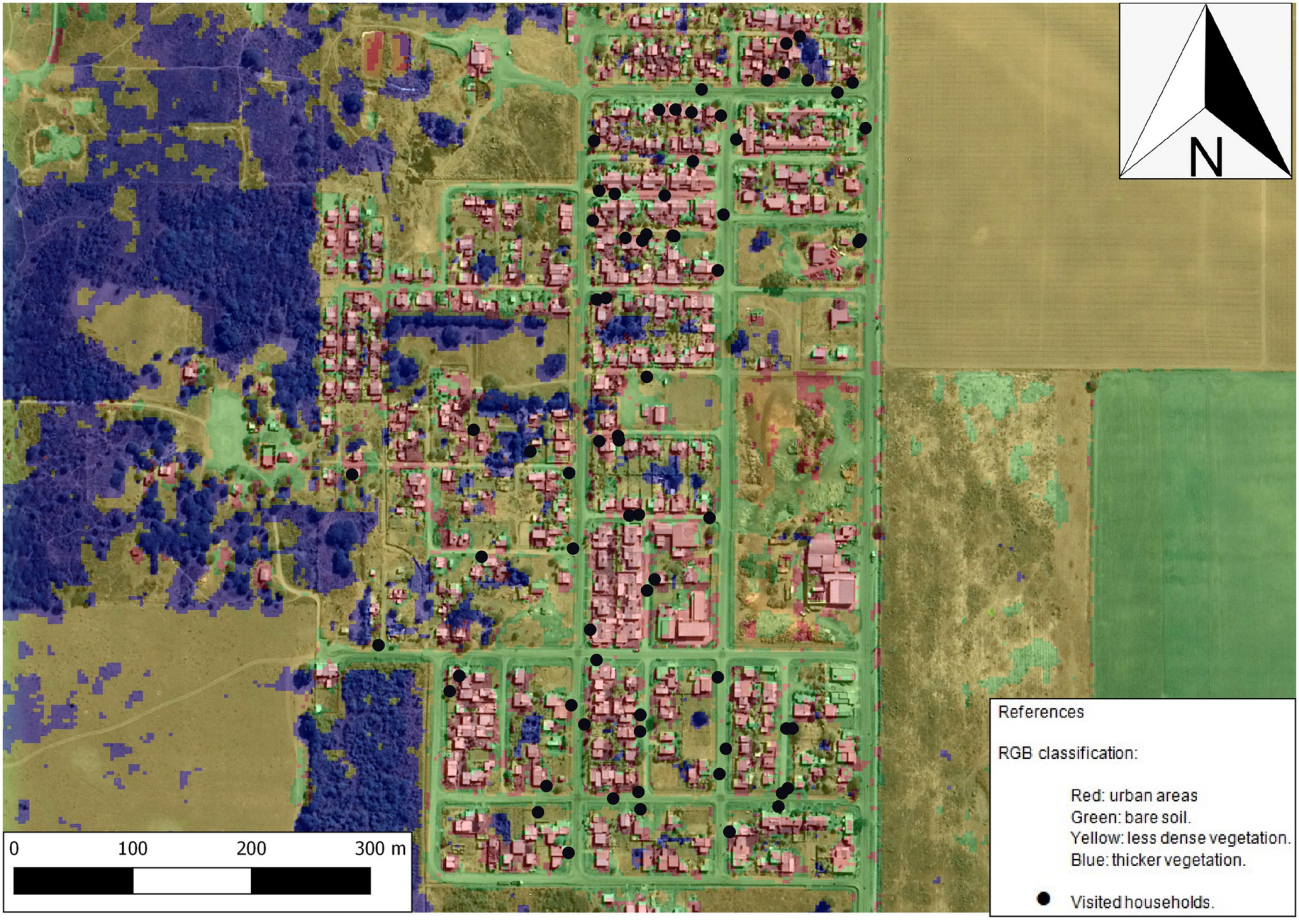
Environmental information derived from RS data: The land-use classification and Landsat environmental indices. Map data © 2020 Google, base map obtained through QuickMapServices QGIS plugin—QGIS Geographic Information System. Open Source Geospatial Foundation Project. http://qgis.osgeo.org. Contains information from OpenStreetMap and OpenStreetMap Foundation, which is made available under the Open Database License.

The analysis of the study area with respect to the environmental variables (Fig 2) shows that the most frequent land cover class was C2 (low and sparse vegetation), followed by C3 (bare soil) and some isolated patches of C1 (bushy or thicker vegetation). This analysis also shows that most of the households were located adjacent to or on bare soil (C3) or areas with urban construction (C4). Table 3 shows the association between the environmental variables around the households with and without individuals infected with IPs. NDWI was the environmental variable more closely associated with infected individuals (p = 0.05); showing lower humidity around their homes.

**Table 3:** Summary of the environmental characteristics in the immediate vicinity of the households with and without individuals infected with intestinal parasites from Parque Industrial, Pampa del Indio, Chaco.

	Environmental characteristics in the immediate vicinity of households with infected individuals	Environmental characteristics in the immediate vicinity of households without infected individuals	p-value for the difference in means
**Mean of NDVI [Me.(SD)] [CI 95%]**	0.388 (0.005) [0.377–0.400]	0.400 (0.003) [0.393–0.408]	0.08
**Mean of NDWI [Me.(SD)] [CI 95%]**	0.034 (0.004) [0.026–0.043]	0.044 (0.002) [0.038–0.049]	0.05
**Mean of NDSI [Me.(SD)] [CI 95%]**	-0.374 (0.002) [-0.379- -0.368]	-0.372 (0.001) [-0.375- -0.369]	0.69
**Mean of NBRT [Me.(SD)] [CI 95%]**	0.957 (0.0006) [0.956–0.958]	0.958 (0.0003) [0.957–0.958]	0.20
**Mean of C1: urban areas [Me.(SD)] [CI 95%]**	0.340 (0.012) [0.316–0.364]	0.322 (0.007) [0.310–0.341]	0.30
**Mean of C2: bare soil [Me.(SD)] [CI 95%]**	0.295 (0.008) [0.278–0.312]	0.308 (0.006) [0.295–0.321]	0.24
**Mean of C3: vigorous vegetation [Me.(SD)] [CI 95%]**	0.054 (0.006) [0.041–0.066]	0.053 (0.004) [0.045–0.062]	0.96
**Mean of C4: less dense and sparse vegetation [Me.(SD)] [CI 95%]**	0.310 (0.008) [0.293–0.327]	0.312 (0.005) [0.301–0.323]	0.85

Me.: Mean; SD: Standard Deviation; CI: Confidence Interval; NDVI: Normalized Difference Vegetation Index; NDWI: Normalized Difference Water Index; NDSI: Normalized Difference Snow Differential Index; NBRT: Normalized Burnt Area Index. Land cover classes: C1 = bushy or thicker vegetation, C2 = low and sparse vegetation, C3 = bare soil and C4 = urban construction.

**Fig 3:**
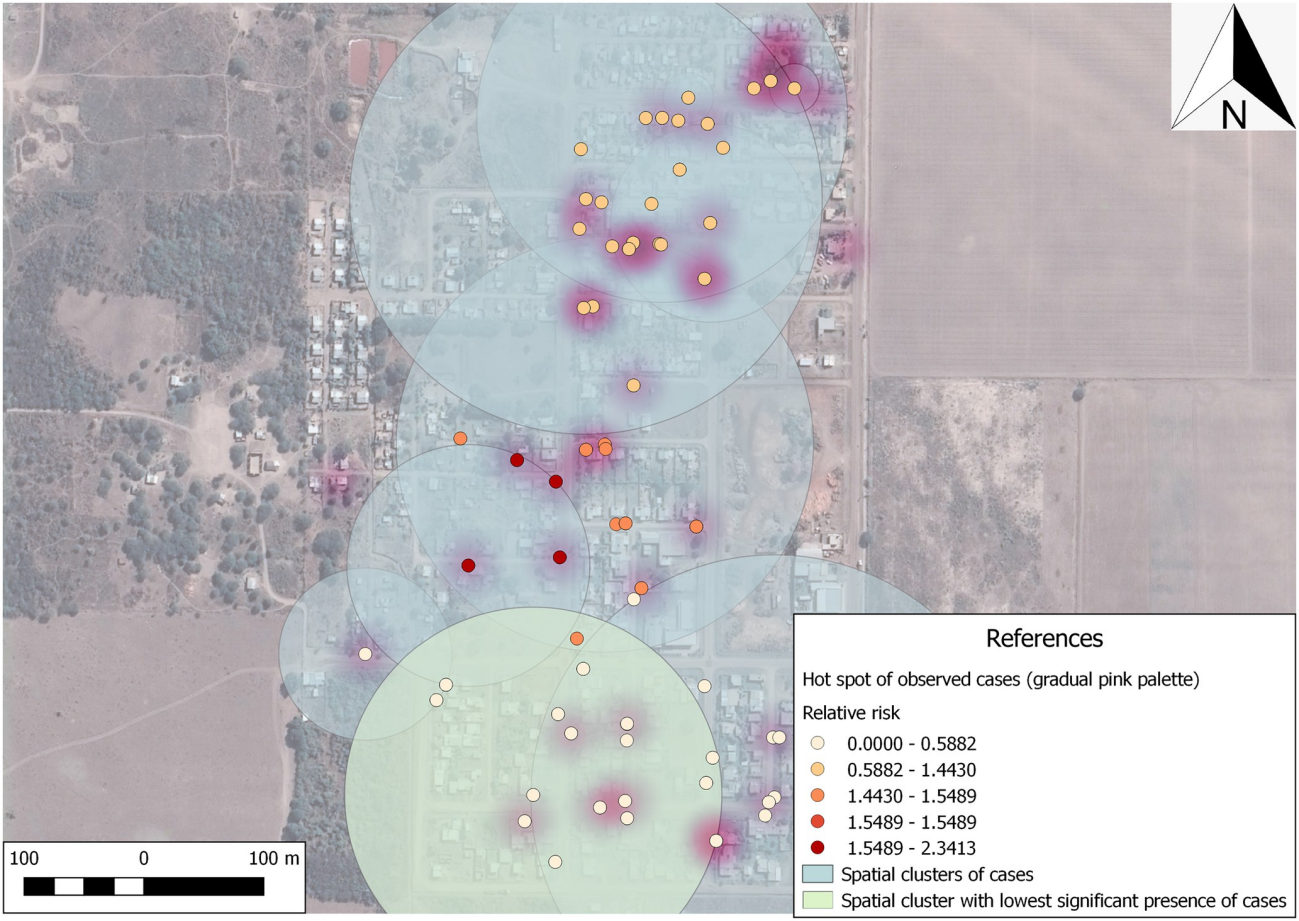
Purely spatial pattern analysis performed with SaTScan^™^ software, version 5.1.3. Map data © 2020 Google, base map obtained through QuickMapServices QGIS plugin—QGIS Geographic Information System. Open Source Geospatial Foundation Project. http://qgis.osgeo.org. Contains information from OpenStreetMap and OpenStreetMap Foundation, which is made available under the Open Database License.

For the spatial analysis, a total of 69 households were included given that not all households were geolocalized, this comprised a total of 302 inhabitants. This analysis showed a spatial pattern of clustering in a north-south direction, with a higher proportion of positive individuals in the northern part of the neighborhood. The output of the spatial cluster analysis presented one significant cluster covering an area of 0.16 km, including 17 households with 73 individuals. In this cluster, according to the quasi maximum likelihood method, there should be 23 individuals with IPs, but the number of observed cases was actually 11, thus determining a ratio of 0.48 and establishing a relative risk of 0.41 with a spatial prevalence of 15.1% (Fig 3). The spatial cluster with the lowest significant presence of cases (in light green) is observed in Fig 3, containing a greater proportion of houses with fewer relative risk. These houses within the low risk cluster for IPs had a significantly higher NDVI (*p* < 0.01) and NDWI (*p* < 0.01) than those houses outside the cluster; this is probably due to the large adjacent area to the left of the cluster presenting thicker vegetation. With respect to the land use classes, the houses within this lower risk cluster fell under class C2 (low and sparse vegetation (*p* < 0.01).

The analysis of the variables and the output of the logistic regression (Table 4), shows the crude odds ratios (ORs) that were statistically significant or close to significance in the bivariate models and their 95% confidence intervals (CI). Both income from a retirement fund or pension, as well as age were significantly associated with the absence of IPs, supporting the presence of these variables in a subsequent multivariate logistic model. As observed, those individuals older than 18 years had 0.6 times less chance (protective factor) of presenting IPs in comparison to children and adolescents. Similarly, a person receiving a retirement fund or pension had 0.44 times less chance of being infected with IPs. These two variables, age and pension fund, showed a statistically significant adjusted interaction (OR, *p* = 0.03), showing to be a protective factor, since the risk of infection with IPs is decreased by 0.24 (95% CI: 0.06–0.87). The environmental variables NDWI and NDVI were also included in the multivariate regression given their near significance (*p* = 0.05).

**Table 4:** Risk of intestinal parasites: Bivariate logistic models at the individual level in a neighborhood from Pampa del Indio, Chaco (Argentina) during 2016–2017.

	OR	P-value	CI (95%)
**Household income from retirement fund or pension**	0.44	0.04	0.18–0.99
**Age (years): Adults (18 or more)**	0.65	0.03	0.39–0.95
**NDWI: Normalized Difference Water Index**	0.002	0.05	0.002–1.02
**NDVI: Normalized Difference Vegetation Index**	0.02	0.05	0.0002–1.08

OR: Odds Ratio; CI: Confidence Interval.

The negative binomial regression model, using the number of infected individuals per household as the independent variable, presented very good performance metrics (prob >Chi2 = 0.000; Pseudo R2 MacFadden = 0.239; R2 = 0.56; Prob >LR = 0.02) and included 9 of the 16 variables collected in the study as predictor variables (Table 5). The model predicts a mean of 1.4 infected per household (SD +-0.5, max: 8 persons), similar to the real values. A normal and cero-centered distribution of residuals was obtained. This result can be understood as a complementary validation of the model´s performance to the analyzed dataset.

**Table 5:** Components and influence of the negative binomial risk model (number of infected individuals per household) in a neighborhood of Pampa del Indio, Chaco (Argentina) during 2016–2017.

Predictor variables	Modification in risk prediction of people infected per household
**Parasitological variables**	
Previous infection	decreases
Previous parasitic treatment	decreases
**Socioeconomic variables**	
Economic income (day laborer)	decreases
Building characteristics (ceramic floor)	decreases
Building characteristics (dirt floor)	increases
**Environmental variables**	
NBRT (proxy of temperature)	decreases
Class 1 (urban area)	decreases
Class 2 (bare soil)	decreases
Class 3 (dense vegetation)	decreases
Class 4 (low and sparse vegetation)	increases

NBRT: Normalized Burnt Area Index

## Discussion

In this study, a public health problem, the presence of IPs, was addressed using RS as a novel tool in a neighborhood of Pampa del Indio, Chaco (Argentina); evidencing the useful approaches these geospatial tools can provide to landscape epidemiology for health [39–43]. There is a lack of data on the prevalence of IPs in Chaco province, except for two small studies conducted in Resistencia, the capital city, more than 18 years ago [66, 67], and another study conducted in rural areas, adjacent to Pampa del Indio, in 2018 [21]. These previous studies found IPs, both protozoan and helminths species, while STH were found only in the studies from Resistencia. Therefore, this is the first report of the presence of IPs in the city of Pampa del Indio.

The total prevalence of IPs found in this study was 30.6% (n = 96), with 35 individuals presenting multiple infections (11.1%). Two of the most prevalent parasites were *G*. *lamblia* (12.7%, n = 40) and *H*. *nana* (7.6%. n = 24). Overall, the prevalence of STH was low, with a total of 8 participants infected with *S*. *stercoralis* (2.5%), this species was also detected in the studies from Resistencia with similar prevalences, although in those studies, they also detected hookworm, *A*. *lumbricoides* and, to a lesser extent, *T*. *trichiura* [66, 67]. Given that a specific method for *S*. *stercoralis*, such as agar plate or Baermann, was not used, it might have been underdiagnosed. Moreover, since this STH can be maintained in an individual for many years due to autoinfection and lack of treatment, these individuals might have gotten infected elsewhere.

The low prevalence of STH in this region of Chaco, as evidenced by the current study and a study conducted in adjacent rural areas [21], might be related to the environmental variables of the area, which, as observed in a previous study from Santiago de Estero (Argentina), are not conducive to the maintenance of the infective stages of these parasites in the soil [57]. Despite the presence of improved water and sanitation in the neighborhood studied, the individuals had a 30.6% prevalence of other IPs, evidencing the presence of fecal contamination, yet the only STH detected was S. stercoralis. Regarding the land use at the immediate vicinity of individual households, a higher proportion of people infected with IPs (except STH) live in an area with sparse vegetation and with households located adjacent to or on bare soil or urban area. The average Normalized Difference Vegetation Index (NDVI) and Normalized Difference Water Index (NDWI) in the vicinity of those dwellings was 0.39, and 0.03 respectively; both values are lower than vicinities without IP infections. This could explain the low prevalence of STH which are more sensitive to the external environment. Previous studies have shown that the characteristics of the area we describe in this study are not conducive to the development of STH with respect to precipitation, soil, temperature, and vegetation cover [43, 68–77]. The environmental characteristics of the study area and the presence of improved latrines in 85% of the households seem to act as protective factors with respect to STH infection.

The use of a detailed resolution of 0.5 m for the land use product, proved to be useful for the risk analysis presented herein (Table 5), since it allowed visualization of the spatial co-occurrence of four land covers (urban, bare soil, as well as both sparse and vigorous vegetation) in a very small peri-urban area. The analysis showed that all four classes were significantly associated with the number of infected individuals per household, allowing the study of the environment-parasite relationship at a very fine scale.

Although the prevalence of STH was low, the presence of other IPs is evident, especially those which are waterborne, like *Giardia* spp., and those that can be transmitted directly from person to person, i.e. *H*. *nana*. Moreover, the prevalence of infection between children and adults was significantly different, being higher in children; individuals of adult age had 0.65 times less chance of presenting an intestinal parasite in comparison to children and adolescents. This agrees with previous reports [51, 57, 78, 79] and is linked to behavioral differences between children and adults, since they are more likely to play in the soil and have lower hygienic standards [80–83]. Additionally, the socioeconomic characteristics of the dwellings and the physical environment (economic income and floor type) were also associated with the number of infected individuals in this neighborhood from Pampa del Indio which is composed mostly of material houses in a peri-urban area, with access to a water network, an electrical network and latrines.

Access to clean water and sanitation has been previously associated with IP infection [84–90]. In this study area, even though all the participants declared obtaining drinking water from the public water network, there were many individuals infected with amoebae and/or *Giardia* spp., evidencing either a sub-optimal quality of the water for drinking and cleaning of fruits and vegetables, due to either contamination from the water treatment plant to the households, contamination during storage of water in the household, or to improper functioning of the water treatment plant. The infection with *Giardia* spp. could also be due to zoonotic genotypes, as previously found in an area from Puerto Iguazú (Misiones, Argentina) [79] and given that many participants had domestic animals in their households. Nonetheless, this aspect escaped the aim of the current study.

The geospatial data and tools used in this study proved to be useful for the study of the relationship between the different factors that influence the presence of IPs in a community, from an eco-health or one-health approach. Moreover, most of the geospatial data and tools used are freely available and obtained by RS; this is very relevant in the field of epidemiology when it comes to balancing resources spent on data collection in the field. The risk analysis model used in this study shows that the dimension that most influences the prediction of the model are the environmental variables, specifically those coming from the classification carried out to validate the criteria established to differentiate the classes. Precisely these environmental characteristics (soil, vegetation, and humidity) have been previously associated with the presence of STH in Argentina at the national scale [43].

A limitation of this study, although the sample size was representative of the neighborhood Parque Industrial of Pampa del Indio, is that it may not be extrapolated to the entire city; therefore, the results were worked at a very small scale, both at the household level and spatially. As mentioned above, another limitation specific for the presence of *S*. *stercoralis* is that a coprological technique specific for this parasite was not used and therefore the prevalence might be underestimated.

## Conclusion

Most of the IPs found in this study were of water-borne transmission and those transmitted directly from person-to-person which is usually associated with quality of the water used for drinking and food manipulation, hygiene and overcrowding. There was a low prevalence of STH in this area, despite a moderate prevalence of other IPs, with only a few cases of *S*. *stercoralis*. We believe that this is related to the environmental characteristics of the area, which are unsuitable for the development/permanence of the infective stages of STHs in the soil, given that deworming is not routinely performed in this area and fecal contamination is occurring.

The spatial analysis of the IPs found showed a marked north to south distribution of IPs with a predominant low-risk cluster in the south. Given that sometimes it’s difficult to obtain large scale epidemiological data, it is relevant to generate models with local and geospatial data that can learn from free and open access data sources to estimate an accurate probability of occurrence, providing a better understanding of risk and prioritizing situations of greater vulnerability for evidence-based, strategic, and effective implementation of public policies in general, and health policies in particular.
